# Analysis and Optimization of Bulk DNA Sampling with Binary Scoring for Germplasm Characterization

**DOI:** 10.1371/journal.pone.0079936

**Published:** 2013-11-19

**Authors:** M. Humberto Reyes-Valdés, Amalio Santacruz-Varela, Octavio Martínez, June Simpson, Corina Hayano-Kanashiro, Celso Cortés-Romero

**Affiliations:** 1 Department of Plant Breeding, Universidad Autónoma Agraria Antonio Narro, Saltillo, Coahuila, Mexico; 2 Genética, Campus Montecillo, Colegio de Postgraduados, Montecillo, Estado de México, Mexico; 3 Laboratorio Nacional de Genómica para la Biodiversidad (LANGEBIO), Centro de Investigación y de Estudios Avanzados del Instituto Politécnico Nacional (CINVESTAV), Irapuato, Guanajuato, Mexico; 4 Department of Plant Genetic Engineering, Centro de Investigación y de Estudios Avanzados del Instituto Politécnico Nacional (CINVESTAV), Irapuato, Guanajuato, Mexico; Nanjing Agricultural University, China

## Abstract

The strategy of bulk DNA sampling has been a valuable method for studying large numbers of individuals through genetic markers. The application of this strategy for discrimination among germplasm sources was analyzed through information theory, considering the case of polymorphic alleles scored binarily for their presence or absence in DNA pools. We defined the informativeness of a set of marker loci in bulks as the mutual information between genotype and population identity, composed by two terms: diversity and noise. The first term is the entropy of bulk genotypes, whereas the noise term is measured through the conditional entropy of bulk genotypes given germplasm sources. Thus, optimizing marker information implies increasing diversity and reducing noise. Simple formulas were devised to estimate marker information per allele from a set of estimated allele frequencies across populations. As an example, they allowed optimization of bulk size for SSR genotyping in maize, from allele frequencies estimated in a sample of 56 maize populations. It was found that a sample of 30 plants from a random mating population is adequate for maize germplasm SSR characterization. We analyzed the use of divided bulks to overcome the allele dilution problem in DNA pools, and concluded that samples of 30 plants divided into three bulks of 10 plants are efficient to characterize maize germplasm sources through SSR with a good control of the dilution problem. We estimated the informativeness of 30 SSR loci from the estimated allele frequencies in maize populations, and found a wide variation of marker informativeness, which positively correlated with the number of alleles per locus.

## Introduction

DNA bulks are valuable for studying large numbers of individuals and markers. Each bulk is a combination of DNA from several individuals, usually up to 30 samples, coming from a given group. The source can be a family, a local population sample, a taxonomical unit, a variety, a set of selected individuals, etc. The analysis of the bulk genotype can be of the type absent/present band scoring, or based on estimation of allele frequencies by band intensity.

Bulked segregant analysis was developed to find markers linked to a given trait [1]. Two sets of individuals are selected from the segregant progeny of a cross, being chosen from the opposite extremes of the phenotypic range of a trait. DNA is isolated from both mixtures, and a given set of marker loci is assayed on both bulks. This approach helped the authors of this method to identify marker loci linked to downy mildew resistance in lettuce, by scoring absence or presence of bands on the electrophoretic patterns generated by the RAPD approach. The same approach was used to find a SCAR marker for early sex determination in *Carica papaya*, through RAPD analysis of bulked DNA from 25 hermaphrodite and 25 female plants [2]. Bulk DNA sampling has been extended to cases that do not involve planned crosses. For example, an AFLP marker strategy was used on 10 male and 10 female bulks from collected individuals of the tree *Eucommia ulmoides*, a dioecious plant, to develop a SCAR type marker for early sex identification [3]. Polymorphic loci between male and female bulks were validated through the analysis of 20 plants from each sex. Also, in the algae *Gracilaria gracilis*, sex-linked PCR markers were identified from male and female bulks [4]. With the aid of bulked DNA analysis in maize landraces, the *Dwarf*8 gene was found to be involved in climatic adaptation through diversifying selection for flowering time [5].

DNA pooling is useful in large scale association studies to reduce the cost of analyzing resistance and susceptibility genes for several diseases [6]. In human genetics pooling was first used for an association study in type I diabetes mellitus [7]. The pooling approach was also used in the identification of a Bardel-Biedl syndrome locus in humans [8]. An effective use of DNA pooling for mapping traits might be in a two-stage design, where putative genes identified through bulks are validated through individual genotyping [6]. Highly homozygous cultivars allow an association mapping approach based on a single sample per variety. Through this sampling scheme and a linear mixed model approach, six main effect QTL for ordinal traits were identified from 257 soybean cultivars [9].

Bulk DNA methods are widely used for assessing diversity in plants. For example, this approach has been employed for RFLP diversity estimation among maize populations, allowing the evaluation of allele frequencies from band analysis in three independent balanced DNA bulks of ten individuals randomly sampled from 65 populations [10]. Bulk DNA analysis was also used to study the introduction of temperate maize to Europe based on SSR markers [11]. However, loci had to be filtered for suitability of estimation of allele frequencies, based on correlation with true allele frequencies from SSR assays in line mixtures, selecting approximately 50% of the tested loci. Also for maize populations, a comparison was made between individual genotyping and the bulk approach to estimate SSR allele frequencies in European flint maize populations [12].

A simpler approach for bulk DNA analysis of diversity and for cultivar identification is the use of balanced pools for marker assays, followed by binary scoring of absence or presence of bands. This strategy was used to study nine germplasm sources of alfalfa with AFLPs [13]. The bulks were composed of 30 plants from each population under study, producing binary scores for 34 primer combinations. Binary data were used to estimate genetic distances based on the Jaccard' s coefficient of similarity. The UPGMA cluster analysis revealed hierarchical patterns among the nine germplasm sources, associated with their geographic, subspecific, and intersubspecific hybrid origins. In a similar way, 96 safflower accessions were characterized through AFLP markers in DNA bulks isolated from groups of 12 plants per entry [14]. Electrophoretic patterns were scored in a binary fashion, and data were analyzed with distances based on the proportion of unmatched markers and the UPGMA algorithm. In this way, AFLP markers distinguished safflower populations and revealed that the genetic structure was different between regions. The bulk DNA approach with binary scoring has also been applied to SSR markers. The genetic diversity among 54 maize landraces from Southwestern China was assessed through 42 microsatellite loci in bulked DNA from samples of 15 plants [15]. The UPGMA clustering supported the hypothesis that maize landraces in Southwestern China were initially introduced from India into Sichuan. The study also showed that, although the bulk DNA sampling analysis partially masked genetic diversity among landraces, it was effective to evaluate their genetic relationships. In rice, a bulk-based microsatellite analysis with binary scoring and pools composed of five plants allowed a study of genetic diversity associated to agronomic traits in Pakistani landraces [16].

Although bulk DNA sampling with binary scoring has been successfully used in several applications to characterize populations and to study diversity, a theoretical study is necessary to define optimum sampling strategies and to select informative loci. Variation in sample sizes and sampling strategy has been found in the literature and there is not yet a consensus method to define sampling schemes from preliminary data on allele frequencies.

In the bulked DNA approach, a single DNA fingerprint is obtained in an attempt to characterize, identify or discriminate the given biological unit. From the standpoint of informativeness, two aspects must be considered: (i) the ability to differentiate between a set of populations, i.e. the discriminating power, and (ii) the consistency of DNA fingerprints through replicated sampling, i.e. the opposite of sampling variation, or noise in the jargon of information theory. It would be misleading to evaluate a set of bulk DNA fingerprints for several populations only through a diversity measure such as 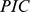, or by its ability to differentiate between units, because hidden noise is not being considered. In fact, it is expected that if the bulk size is reduced, sampling variance will increase, rendering an inconsistent bulk-based fingerprint, albeit with different DNA profiles for different germplasm sources.

In this work we approach bulk DNA fingerprinting for germplasm characterization through binary scoring, with the aim to define: (i) optimum bulk sizes, based on preliminary estimations of allele frequencies, (ii) strategies to deal with the problem of undetected alleles due to small frequency and PCR failure, and (iii) a method for selection of informative markers. Rather than association mapping, the analysis is aimed at optimizing the characterization of accessions and varieties on a large scale in order to monitor changes in polymorphism levels and presence of rare alleles in geographically and genotypically diverse populations in different growing seasons. This would allow better management of local landrace populations and the identification of germplasm which could be incorporated into conservation schemes. The general framework for this research is information theory. This branch of mathematics has been applied in several situations involving genetic markers; for example in the measurement of linkage disequilibrium [17], inference of ancestry [18], SNP selection for association studies [19], [20], statistics for association [21], information for QTL mapping [22] and transcriptome analysis [23]. The Shannon entropy, a key concept in information theory, can be used as a general, firmly mathematically founded, framework for calculating information provided by genetic markers for population and individual identity.

## Methods

### Mutual information between DNA bulk profiles and germplasm sources

Bulk DNA fingerprinting in its simplest form is the conversion of allele frequencies into a binary code resulting from band present-band absent scoring, through the processing of DNA samples from a mixture of plants. We expect that only those allele frequencies above a certain threshold will be likely to produce bands. Thus, the presence of a band indicates that a given allele is present in the sampled population, whereas its absence can be interpreted as absence of the given allele or a lower than threshold frequency in the reference population. From the mathematical point of view, we want to maximize the average information gained about the identity of a given germplasm source in the space of 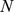 populations, with the knowledge of the band profile of the corresponding bulk. In this work, average information will be based on the foundations of information theory [24]. Let 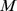 be a random variable with possible values 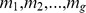 and probabilities 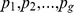, respectively. The Shannon entropy of 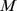 is defined as follows:
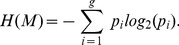
(1)


The expression 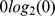 equals 0 by definition. This concept leads to the definition of mutual information, i.e. the information conveyed about a random variable 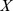 by a variable 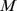, as the average reduction of the uncertainty or entropy of 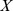 given knowledge of the value of the variable 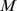 [25]:
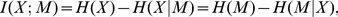
(2)where 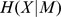 is the average Shannon entropy of 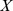, given knowledge of the value of the variable 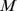. In the context of this work 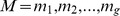 denotes marker genotypes whereas 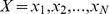 represents germplasm sources. Mutual information in equation (2) can be interpreted as the expected reduction in the entropy or uncertainty about the identity 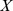 of a random population caused by the knowledge of its marker genotype 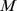. Mutual information is symmetrically defined in terms of entropies; in fact, as we see in in equation (2), it can also be expressed as the expected reduction in the uncertainty about a marker genotype 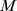 caused by the knowledge of the member identity 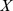. Furthermore, if 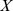 represents either individuals or genetically homogeneous populations then 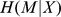 becomes zero, because of null marker diversity within those units. Under this particular situation, mutual information in equation (2) becomes simply 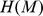, i.e. the Shannon entropy of the distribution of marker genotypes [26].

In DNA bulk fingerprinting for germplasm source identity we want to maximize 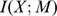. However, if genetic heterogeneity is present within the tested populations and thus sampling error, the term 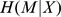 is not assumed to be zero by the fact that there is potential variation in marker genotypes provided by different assays. The term 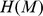 will be called diversity, whereas 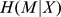 will be called noise. Through optimizing 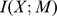 diversity will be increased among the single or multilocus binary profiles of different groups of individuals, while reducing noise in order to carry out a reliable sampling.

To find an analytical solution for an optimal sampling size in bulks, a pattern of distribution of allele frequencies across varieties would have to be assumed. However, a practical approach, which has been used as an example in this work, is to use previous knowledge of allele frequencies in different populations of a species and several loci for a given marker type, and find a consensus sample size that is highly informative. Furthermore, locus selection may be done through estimating the mutual information 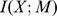. If noise, i.e. the 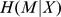 term, which represents the variation among DNA bulks from the same source, can be ignored, then marker informativeness can be calculated simply through the entropy of genotypes among germplasm sources, i.e. 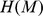. The so-calculated value of information is given in bits, and can be interpreted as the number of fully informative binary loci, or briefly, the effective number of binary loci [26].

### Average bulked DNA marker information per allele

Consider a random bulk with 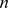 alleles or 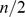 individuals in a random mating diploid population, where the absence or presence of a given allele is scored through a marker system. The maximum information that a given allele can provide is 1, and it depends on the variation of its frequency across germplasm sources and the bulk size. Let 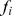, 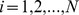, be the allele frequency at the 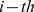 germplasm source. Thus the probability of the allele being absent at the 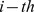 germplasm source is 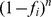 assuming random sampling. If we consider each germplasm source as being equally frequent within a defined set, then the global probabilities of absence and presence of a given allele in a random bulk would be their average probabilities across germplasm sources. Thus the entropy of the binary marker genotypes for a given allele is:
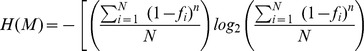


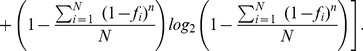
(3)


This is the diversity part of equation (2). The entropy of the marker genotypes conditional on germplasm sources 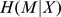, is the average entropy of the binary genotypes across populations:
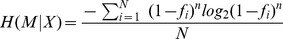


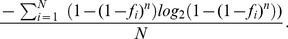
(4)


This is the noise part of equation (2). The information provided by the binary scoring of the given allele is 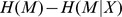. We expect that the value of equation (4) will descend as we increase the bulk size, becoming more replicable. In fact, the limit of 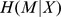 when 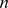 tends to infinity is zero, and information is 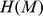.

So far we have considered only the noise due to sampling error. However, there are technical issues that introduce undesirable variation. They are complex and most of them need to be studied on experimental basis [27]. In the case of SSRs, the presence of stutter bands potentially introduces incorrect artificial variation [28]. There are generic problems associated to all electrophoresis-based genetic markers. Two of them are the technical homoplasy, i.e. when two bands are mistakenly considered homologous, and oversplitting, in which bins are too thin and may split variant locations of the same amplicon [27]. Other problems are the occurrence of false positives and false negatives, associated to the so-called bayesian error rates, which represent the probabilities of mis-scoring the presence or absence of alleles. We expect that all the mentioned technical factors reduce the mutual information. For instance, let us consider the case of sensitivity, defined as the probability of an allele being detected given that it is a present in a given bulk. Under the absence of sampling error, if we fix sensitivity to a value of 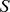, with 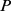 being the frequency of bulks with the presence of a certain allele, then mutual information is modified to 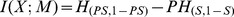, where entropies are calculated with the probability vectors in their subscripts. It can be graphically demonstrated that information is monotonically increasing with sensitivity, reaching a maximum of 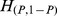 with 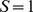 and a minimum of 0 with 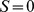. In this work we analyze one aspect of technical noise: the allele dilution effect on its detection.

### The problem of allele dilution

We have assumed so far that any allele present in a bulk will be revealed by the given marker system. However, that is not always the case because it has been observed that, depending on the marker system, a low frequency allele in a bulk may not be detected. For RFLP and RAPD in tomato, it was found that an allele was still detectable at a proportion of 0.05 in a DNA pool [1]. Kuboki, Yoshimura and Yano [29] reported a similar threshold of detection in rice. These reports were confirmed by Dubreuil et al. [10], who found that an RFLP allele is barely detectable when present in a proportion of 0.05 within a DNA pooled-sample.

For AFLPs, the lowest ratio at which an allele was still detected in a range of crop species was 1:24, i.e. with a dilution threshold of approximately 0.05 [30]. However, below the 1:4 ratio only a few bands of high original intensity could still be recovered. For SSR markers in maize, Reif et al. [12] found that alleles in a dilution below 0.2 were most often undetected. For SSR markers, Dubreuil et al. [11] tested dilutions of 0.025, 0.05, 0.10, 0.20, 0.30, 0.40 and 0.50 for inferring allele frequencies from DNA bulk data. From the optimal SSR markers they found a range of correlations from 0.79 to 0.99; however they did not report a specific detection threshold.

In view of the above information, we considered a range of threshold dilutions from 0.05 to 0.2 in order to evaluate a strategy for coping with the dilution problem. The selected strategy to overcome the technical problem of rare allele amplification was the use of fractionated bulks, in the same way as the strategy used by Dubreuil et al. [10] for genotyping 10 maize populations from North America, where three independent bulks of 10 plants were randomly selected from each population.

Let 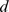 be the threshold dilution for a given marker, this being the minimum dilution from which an allele can be detected in a bulk of 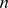 alleles randomly sampled from a population with allele frequency 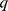. The probability of the allele being undetected in a bulk is:
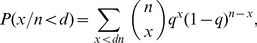
(5)where 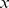 is the number of copies of the allele in the bulk. The size of the bulk is equivalent to 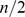 plants from a random mating population. If the sampling strategy consists in 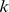 independent bulks of sample size 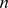, the probability of the allele being undetected is 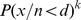. The scoring scheme under this strategy would be to assign the symbol 1 if the allele shows up in at least one of the 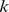 bulks, and 0 otherwise.

To further check the problem of allele dilution and to validate the tested range, we assayed three SSR loci for corn: phi093, phi072 and phi064. The biological material comprised three maize accessions collected in the state of Puebla in Mexico. The plants were analyzed both individually and in fractionated samples ranging from 22 to 30 plants, divided into bulks of four to 10 plants. The number of different SSR alleles in those samples ranged from 3 to 10. PCR reactions were carried out using standard protocols and run on a 3730 ABI DNA ANALYZER. Scoring was carried out with the GeneMapper Software Version 4.0 (AppliedBiosystems).

### Bulk DNA sampling with SSRs across maize populations

As an example application of the model, a set of 56 maize populations was sampled by selecting 10 random plants per germplasm source, which were individually genotyped using microstatellite markers, called SSRs. The source of this genetic material was the active collection of the USA maize germplasm bank, curated by the USDA in the North Central Regional Plant Introduction Station (NCRPIS) at Ames, Iowa. These populations were collected in USA, Mexico, Argentina, Peru, Brazil, Chile, Ecuador, Paraguay and Uruguay. A total of 31 SSR primers were used for microsatellite amplification. Genomic DNA was extracted from leaves of young maize seedlings by using the commercial PUREGENE DNA Isolation Kit [31]. The PCR products were separated and visualized in polyacrylamide gels (4% denaturing 6 M urea, 29:1 acrylamide:bisacrylamide), and a file containing the information for each gel was generated by the ABI GeneScan software. The gel files generated by the ABI GeneScan software were scored using the computer software STRand [32] following the local Southern algorithm for estimating allele sizes. The 31 SSR assays resulted in 198 polymorphic bands. These data have been submitted to USDA; part of them are already in the page http://www.ars-grin.gov/npgs/(Accessed 2013 September 2) and the remaining are in process of being curated.

A total of 191 of the 198 alleles identified in this study were selected for optimization, since complete data sets were available for these alleles. Equations (3) and (4) were written as R functions [33] to estimate diversity, noise and information for bulk sizes of 10 to 100 alleles, with increments of 10. Plots were exhaustively examined to see the trends across increasing bulk sizes, and an average plot was constructed.

To examine the dilution problem, a sample of 60 alleles divided into 1, 2, 3, 5, 6, 10, 15 and 30 bulks, was evaluated for the probability of a given allele being undetected, by equation (5). These numbers of divisions of the sample were restricted to generate bulks with an integer number of plants, i.e. 30, 15, 10, 6, 3 and 1, respectively. The first case is the situation when the 60-allele sample is not divided. The last case represents the genotyping of 30 individual plants. The evaluated threshold dilutions were 0.05, 0.1, 0.15 and 0.02. The allele frequencies were set as 0.1, 0.2 and 0.3.

Information for 30 SSR marker loci was calculated through simulated bulks of 30 plants for each of the 56 maize populations, based on the estimated allele frequencies. A multinomial simulation was performed to generate each set of 60 sampled alleles through the *rmultinom* command in R [33]; afterwards, the absence or presence of each allele in the sample was tested, and then encoded as either 0 or 1, respectively, thus generating a vector of size equal to the number of alleles of the given SSR in all populations. After bulk simulation for each SSR locus, the entropy of the electrophoretic patterns, coded by binary vectors, was estimated through equation (1). This value of 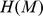 was taken as the amount of information per SSR locus, since we did not consider 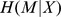 due to the fact that with 60 sampled alleles this term becomes almost zero. One of the SSR loci from the set of 31 was not considered due to missing data.

## Results and Discussion

### Bulk size optimization

**Figure 1:**
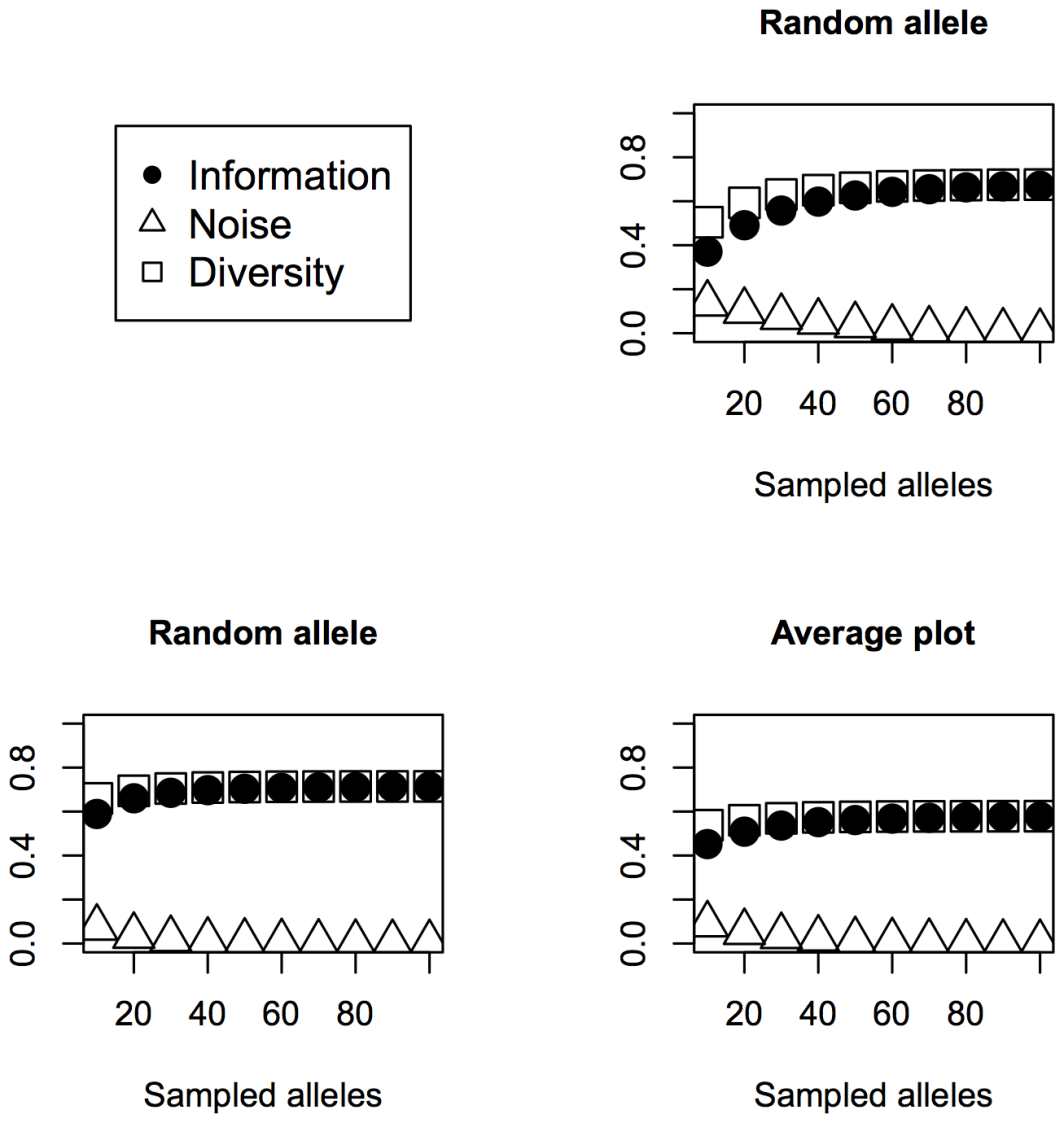
Plots of information parameters for several bulk sizes, based on estimated allele frequencies in maize germplasm sources.

Although the sample size of 10 maize plants per population was small, this survey allowed us to evaluate general tendencies of allele frequencies for DNA bulked SSR marker information in this species. Based on the estimated allele frequencies, Fig. 1 depicts plots of information and bulk size for two random alleles and for the average trend, whereas average values for all sample sizes are presented in Table 1. In general, the results showed that from bulks of 60 alleles or more, the noise term is close to zero, with information reaching a near-maximum level, being almost equal to the diversity term, i.e. the entropy of the binary marker genotypes 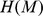 defined in equation (3). The average information per allele for bulks based on 60 alleles, i.e. 30 plants, was 0.568 bits with a diversity value of 0.577, with the noise term oscillating between 0 and 0.048, and a mean of 0.006. The bulks of 100 alleles, i.e. 50 plants, gave the maximum information in the tested range: 0.577 bits with an average noise of 0.002. However, as can be observed in Fig. 1, this does not represent an appreciable advantage over bulks composed of 60 alleles. Thus, at least for maize germplasm sources, our results indicate that bulked DNA from 30 plants can provide a close to optimum information per SSR allele.

**Table 1:** Average values for information parameters in bulks of 10 to 100 SSR alleles in maize.

Parameter	10	20	30	40	50	60	70	80	90	100
Diversity	0.538	0.560	0.569	0.574	0.576	0.577	0.578	0.579	0.579	0.579
Noise	0.086	0.052	0.033	0.022	0.014	0.01	0.006	0.004	0.003	0.002
Information	0.452	0.509	0.536	0.552	0.562	0.568	0.572	0.574	0.576	0.577

The drawback to this recommended sample size is that for large bulks, e.g. with more than 20 alleles, the dilution problem must be considered by the fact that alleles with a very low frequency within the bulk may not be detected due to insufficient sensitivity of the marker protocol.

### Strategy for the problem of allele dilution

**Figure 2:**
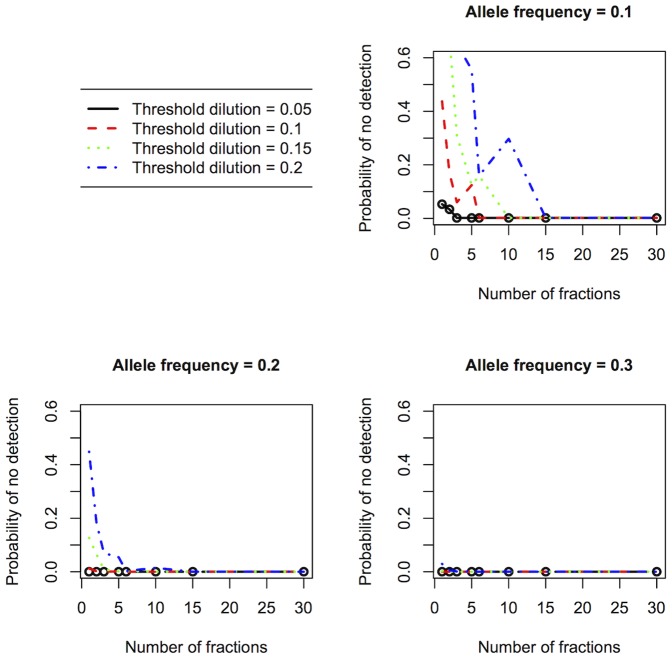
Probability of an allele being undetected for different numbers of fractions of a 60-allele sample, under several dilution thresholds and population allele frequencies.

We have already found that 60 allele bulks sampled for SSR markers in maize provide a near-optimum amount of information; the question now is what would be an adequate number 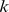 of independent bulks, such that 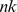  =  60, to cope with the dilution problem. Fig. 2 depicts results for different threshold dilutions and population allele frequencies. For an allele frequency of 0.1 the ability of detection with a threshold dilution of 0.05 performs well, and almost perfectly for 60 alleles divided into three bulks or more. For a threshold dilution of 0.1, division into three bulks still works well, giving a probability of non-detection less than 0.1. However, for an allele frequency of 0.1, threshold dilutions of 0.15 or more make the bulk system unreliable, even dividing the 60 allele sample into six bulks of 10 alleles. The oscillation observed for threshold dilutions of 0.1 and more, although apparently illogical, has an explanation based on the minimum number of copies being present in a given bulk, which can be detected. For example, let us compare three and five divisions for a threshold dilution of 0.1. In the first case, the bulks are composed of random samples of 20 alleles; thus, two or more copies of the rare allele are required to be detected, which has a probability of failure of 0.392 for a single bulk and 0.06 for the three bulks. On the other hand, if we use five bulks of 12 alleles, those samples also need at least two copies of the rare allele, having a probability of failure of 0.66 for a single bulk and 0.124 for the five bulks. In other words, the oscillation obeys the discrete nature of the required minimum number of copies of the rare allele to be detected.

For an allele frequency of 0.2, even under a threshold dilution of 0.2, 60-allele samples divided into three or more bulks perform well, with probabilities of non-detection always lower than 0.1. For allele frequencies of 0.3, even undivided samples of 60 alleles perform well. Thus, in general, we can say that, for random mating maize populations genotyped with SSRs, samples of 30 plants fractionated into three bulks of 10 plants are reliable for rare allele detection with allele frequencies of 0.1 or higher, for threshold dilutions of 0.1 or less. Such was the strategy used by [8] in bulked analysis of maize populations. For threshold dilutions above 0.1, failure of detection of rare alleles must be taken into account, according to equation (5) and the tendencies depicted in Fig. 2.

The results of the bulked and individual assays for three SSR loci to evaluate dilution effects are summarized in Table 2. The accessions are coded as PL077, PL092 and PL149. The accession keys are followed by the bulk numbers. The rows with bold face characters show the results of pooling the information of the three bulks for each combination of marker and accession. The column of alleles gives their numbers based on individual plant assays. The dilution range was calculated in basis of allele frequencies from individual assays within bulks. Detection percentages were calculated from the fraction of alleles that were amplified in the bulked analyses. The dilutions in undetected cases are the frequencies of the alleles that the bulk-based analyses were unable to detect in each assay. The minimum allele dilution was 0.05, with a maximum of 0.70. In most cases the bulk-based approach allowed detection of all the alleles present in the individual plants. The only exception was the locus phi093, where alleles with frequencies of 0.10, 0.11 and 0.15 were not amplified. However, the combined information of the three bulks within the sample allowed detection of the three present alleles, thus reaching a 100% rate of detection. The primers for loci phi064 and phi072 allowed detection of all alleles through bulk-based analyses, even those with a dilution of 0.05. The presence of multiple allelism, e.g. in bulks PL077—3 and PL149—1 for marker phi064 with nine alleles, did not affect their amplification in the PCR experiments. In all cases, alleles with dilutions of 0.2 or more were detected, with most bulks having a successful threshold dilution of 0.05. These results show the usefulness of dividing samples into bulks of 10 plants.

**Table 2:** Analysis of three SSR loci for their amplification under allele dilution.

Marker	Bulk	NP	Alleles	DR	Detection (%)	DU
PHI093	PL092—1	10	3	0.10—0.70	66	0.10
	PL092—2	10	3	0.15—0.30	66	0.15
	PL092—3	9	3	0.11—0.65	66	0.11
	PL092	29	3	—	100	—
PHI072	PL077—1	10	6	0.05—0.52	100	—
	PL077—2	10	5	0.05—0.55	100	—
	PL077—3	10	6	0.05—0.43	100	—
	PL077	30	9	—	100	—
PHI064	PL077—1	10	7	0.05—0.15	100	—
	PL077—2	10	8	0.05—0.20	100	—
	PL077—3	10	9	0.05—0.20	100	—
	PL077	30	9	—	100	—
	PL149—1	9	9	0.06-0.22	100	—
	PL149—2	9	7	0.06-0.22	100	—
	PL149—3	4	5	0.13—0.5	100	—
	PL149	22	10	—	100	—

NP  =  number of plants, DR  =  dilution range, DU  =  dilution in undetected cases.

The global result for each combined bulk is given in bold face characters.

### Information of SSR loci

**Figure 3:**
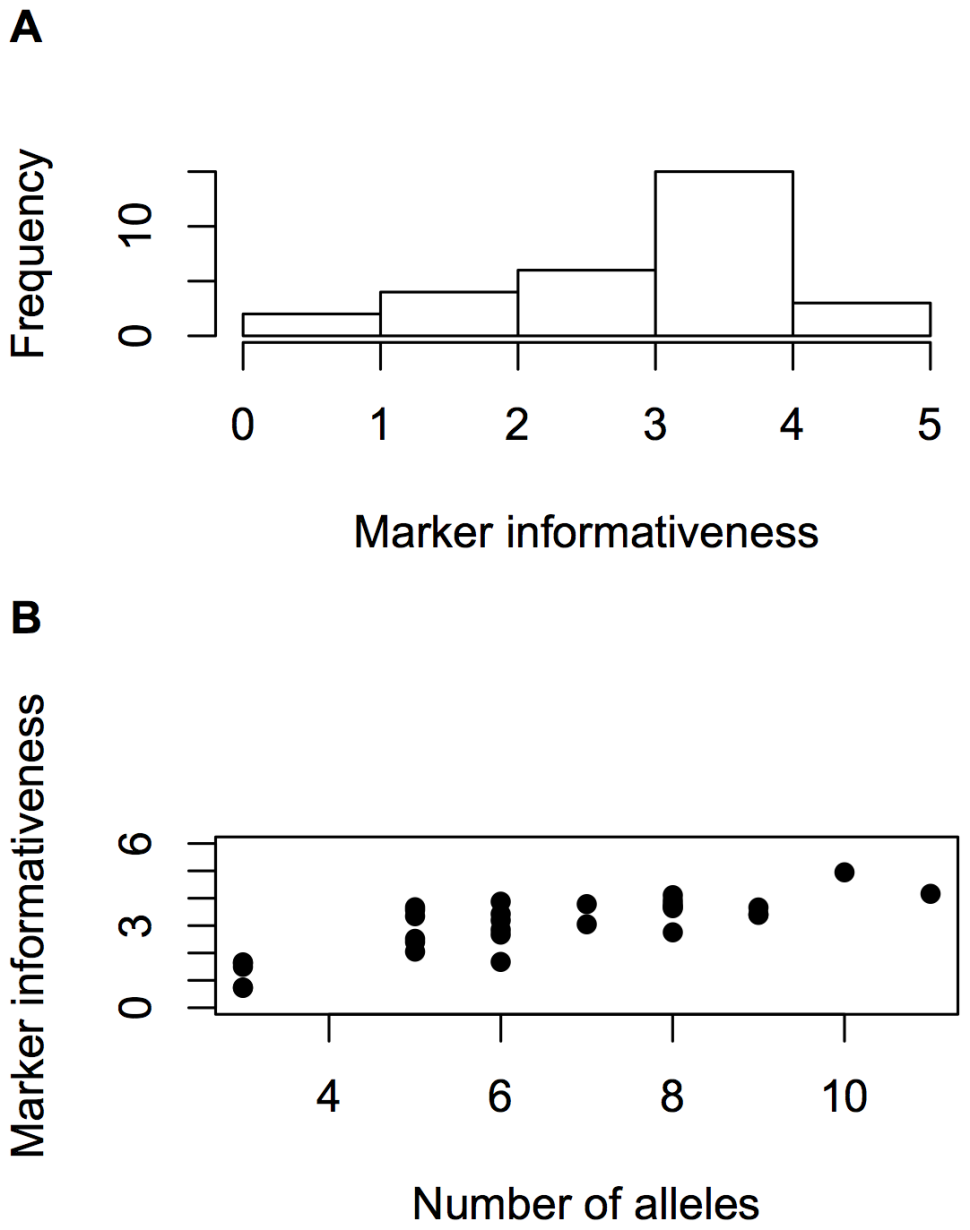
Informativeness of SSRs. Distribution of informativeness in 30 SSR loci measured in bits (a). Association between SSR marker informativeness and number of alleles per locus (b).

Results of the estimation of SSR information are shown in Table 3. The average information per SSR locus was 3 bits, i.e. it was equivalent to 3 binary, fully informative markers, and ranged from 0.795 (phi213984) to 4.951 (phi064), with the highest frequency being placed in the interval from 3 to 4 bits (Fig. 3A). The number of alleles per locus ranged from 3 (phi121) to 11 (phi96100). Information was positively correlated with the number of alleles (Fig. 3B), with a linear correlation 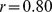, 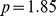 x 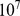 for a two-tailed 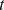 test, and a 95% confidence interval of 0.616 to 0.900, thus indicating that allelic number may work at least as a rough indicator of SSR informativeness.

**Table 3:** Informativeness of SSR loci for bulk DNA genotyping.

SSR	Alleles	Information
phi127	6	3.429
phi051	6	2.677
phi115	3	1.490
phi015	6	3.143
phi033	6	2.848
phi053	8	3.693
phi072	7	3.044
phi093	5	3.609
phi024	7	3.820
phi085	8	2.693
phi034	8	3.904
phi121	3	0.667
phi056	8	3.758
phi064	10	4.951
phi050	3	1.627
phi96100	11	4.130
phi101249	8	4.128
phi109188	9	3.662
phi073	5	3.378
phi96342	5	2.390
phi109275	6	3.910
phi427913	9	3.409
phi265454	8	3.640
phi402893	9	3.288
phi346482	5	2.058
phi308090	5	2.544
phi330507	6	1.796
phi213984	3	0.795
phi339017	3	1.664
phi159819	5	3.578

The goal of calculating individual marker information is to have a criterium for locus selection; such as in the case of the choice of SNP markers in association studies [19], [20]. However, the advent of high-throughput technologies and informatics tools make possible to analyze simultaneously a high number of markers and select the subset related to a given trait [34]. This work however is not aimed at association, but rather the optimization of the necessary number of informative markers to enable rapid large scale population monitoring.

### Suggestions for bulk sampling optimization

For bulk sampling with binary scoring with SSRs in corn, we recommend using 30 plants per accession, divided into three pools of 10 plants. Comparing allele presence among the three bulks of 10 plants per accession, will give an idea of the robustness of allele detection. The cases were results are consistent among the three bulks would be instances with low genotyping error. When only one of the bulks allows detection of a given allele whereas the others give a negative value, the detection of this allele may be subject to a high sampling error or technical problems. Furthermore, validation of the method requires individual genotyping in a fraction of the sampled accessions.

When working with a different species or marker system, previous allele frequency data for a certain number of accessions can be utilized to check the effect of sampling size on mutual information and its components of diversity and noise for several loci and alleles. This is done by calculating the result of equation (3) minus the one of equation (4). To facilitate calculation, the following R code [33] can be used:

#Basic functions for entropy

MyLog2p<-function(x){if(x =  = 0) 0 else x*log(x,2)} entropy<-function(x){-sum(sapply(x,MyLog2p))}

#Calculator of information, diversity and noise

InfoBulkBinary<-function(p,n)

{n<-2*n;v1<-(1-p)ˆn;v2<-1-v1;mymat<-cbind(v1,v2); div<-entropy(c(mean(v1),mean(v2)));a<-NULL;

for (i in 1:length(p)){a[i]<-entropy(mymat[i,])};

noise<-mean(a);c(div,noise,div-noise)}

#p is the vector of frequencies of a given allele across populations

#n is the bulk size in number of diploid plants.

As an example, for a vector of allele frequencies in 10 accessions: 0.1,0,0.4,0.3,0,0.25,0.5,0.23,0.15,0, the information for a bulk of 20 plants is calculated as follows:

InfoBulkBinary(c(0.1,0,0.4,0.3,0,0.25,0.5,0.23,0.15,0),20)

The result:

[1] 0.88327703 0.01279625 0.87048078,

is given in the following order: diversity, noise and information.

## Conclusions

The information theory perspective provides a practical tool for measuring and optimizing information for genetic markers and it accounts for sampling variation. The concept of mutual information allows consideration of two aspects of marker informativeness in bulk DNA sampling of heterogeneous germplasm sources: the ability to detect diversity among populations and the noise resulting from random sampling within those populations. Data obtained from SSR markers in maize allowed us to infer that DNA bulks of 60 alleles, or 30 plants in a population under Hardy-Weinberg equilibrium, attain a near-maximum information per allele and have a sampling noise close to zero. Divided bulks can help to overcome the problems of DNA amplification resulting from allele dilution, and a practical approach would be to use samples of 60 alleles per cultivar, fractionated into three bulks of 20 alleles, i.e. three pools of 10 plants in random mating maize populations. The analysis of three SSR markers with bulks and individual plants confirmed the usefulness of this approach. We found a wide range of marker informativeness for 30 SSR loci, which was correlated with the number of alleles. The approach herein described is useful for planning experiments using the bulk DNA approach, in terms of sampling sizes, number of bulks and locus selection. This would allow better management of local landrace populations and the identification of germplasm for conservation schemes. Although the proposed method was applied to SSR markers in maize, the theory can be applied to any kind of marker system and other species.
